# Very High Dehydroepiandrosterone Sulfate (DHEAS) in Serum of an Overweight Female Adolescent Without a Tumor

**DOI:** 10.3389/fendo.2020.00240

**Published:** 2020-05-06

**Authors:** Daniel I. Iliev, Regina Braun, Alberto Sánchez-Guijo, Michaela Hartmann, Stefan A. Wudy, Doreen Heckmann, Gernot Bruchelt, Anika Rösner, Gary Grosser, Joachim Geyer, Gerhard Binder

**Affiliations:** ^1^Pediatric Endocrinology, University Children's Hospital, Tübingen, Germany; ^2^Steroid Research and Mass Spectrometry Unit, Pediatric Endocrinology and Diabetology, University Children's Hospital, Giessen, Germany; ^3^Institute of Pharmacology and Toxicology, Faculty of Veterinary Medicine, Justus Liebig University Giessen, Giessen, Germany

**Keywords:** dehydroepiandrosterone sulfate (DHEAS), dehydroepiandrosterone (DHEA), tumor, steroid sulfatase, transporter proteins

## Abstract

**Introduction:** An increase of serum dehydroepiandrosterone (DHEA) sulfate (DHEAS) is observed in premature adrenarche and congenital adrenal hyperplasia. Very high DHEAS levels are typical for adrenal tumors. Approximately 74% of DHEAS is hydrolyzed to DHEA by the steroid sulfatase (STS). The reverse reaction is DHEA sulfation. Besides these two enzyme reactions, the DHEAS transported through the cell membrane is important for its distribution and excretion.

**Case Presentation:** We present a female adolescent with overweight and a very high DHEAS. The presence of a DHEAS-producing tumor was rejected using ultrasonography, Magnetic Resonance Tomography (MRT), and dexamethasone suppression. STS deficiency was suspected. Sequence analysis revealed a heterozygous nonsense mutation which predicts a truncation of the carboxyl region of the STS that is implicated in substrate binding. No partial gene deletion outside exon 5 was detected by multiplex ligation-dependent probe amplification. The bioassay revealed normal enzyme activity in the patient's leukocytes. A defect of transporter proteins was suggested. Both efflux [multidrug-resistance protein (MRP)2 and breast cancer-resistance protein (BCRP)] and uptake [organic anion-transporting polypeptide (OATP) and organic anion transporter (OAT) carriers] transporters were studied. Sequence analysis of exons revealed a heterozygous Q141K variant for BCRP.

**Conclusions:** A novel heterozygous nonsense mutation in the *STS* gene and a known heterozygous missense variant in the *BCRP* gene were found. The heterozygous nonsense mutation in the *STS* gene is not supposed to be responsible for STS deficiency. The BCRP variant is associated with reduced efflux transport activity only in its homozygous state. The combination of the two heterozygous mutations could possibly explain the observed high levels of DHEAS and other sulfated steroids.

## Introduction

**Figure 1:**
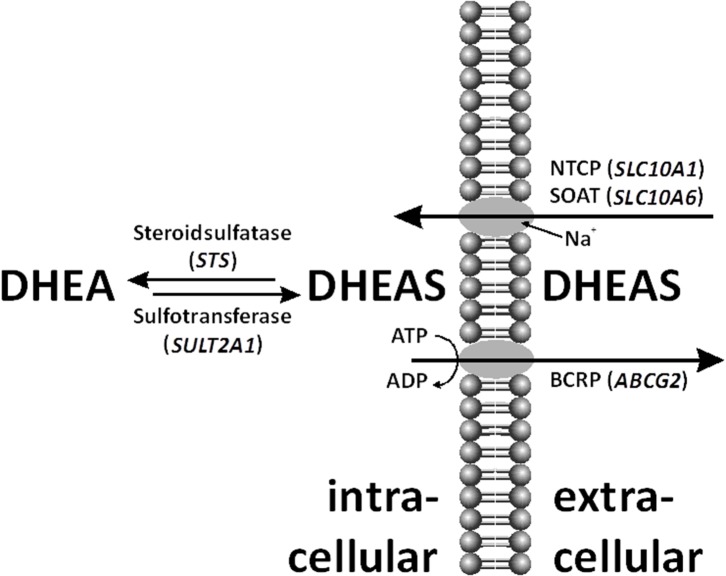
Within the cell, dehydroepiandrosterone sulfate (DHEAS) can be desulfated by steroid sulfatase (STS) and even *de novo* sulfo-conjugated by sulfotransferase (SULT)2A1. DHEAS is shuttled through the cell membrane by various uptake and efflux transporters, such as sodium-coupled cotransporters Na^+^/taurocholate co-transporting polypeptide (NTCP), sodium-dependent organic anion transporter (SOAT), and breast cancer-resistance protein (BCRP).

Dehydroepiandrosterone (DHEA) and its sulfate ester, DHEA sulfate (DHEAS), are prohormones secreted in large amounts by the adrenal zona reticularis. DHEA(S) is the endocrine steroid with the highest concentration in humans. Sulfated steroids were thought to be just metabolic end-products because of their high appearance in the bile and urine. Moreover, sulfated steroids provide a depot for the intracrine release of free steroids at the cellular level. In women, up to 74% of the daily production of DHEAS is hydrolyzed to DHEA (Figure 1) by the steroid sulfatase (STS) (1), and the latter steroid then is converted into biologically active androgens and estrogens.

The human *STS* gene is located in the pseudoautosomal region of the short arm of X-chromosome that escapes X-inactivation (2, 3). Females are carriers of two functional alleles. Males carry one functional and a nonfunctional *STS* pseudogene on their Y-chromosome. Complete deletions or point mutations in the C-terminal half, an area crucial for STS activity, cause X-linked ichthyosis in males (4–6). Affected patients present with large, brown scales and increased thickness of the stratum corneum due to accumulation of cholesterol sulfate (7–9). Females are carriers and with few exceptions do not manifest the disease in the heterozygous state (10, 11).

The reverse reaction is DHEA sulfation (Figure 1). Three different cytoplasmic sulfotransferases, SULT2A1, SULT1E1, and SULT2Bs, are involved in DHEA sulfo-conjugation. SULTs require 3′-phosphoadenosine-5′-phosphosulfate (PAPS) for acquisition of catalytic activity. Lack of DHEA sulfation activity causes hyperandrogenism in females (12).

The transport of the negatively charged DHEAS molecule through the cell membrane by uptake and efflux carriers is important for its distribution and excretion (Figure 1) (13). Steroid sulfate transporter proteins belong to two families: uptake transporters of the solute carrier (SLC) family and efflux transporters of the ATP-binding cassette (ABC) family. DHEAS influx is mediated by several organic anion-transporting polypeptides (OATP1A2, OATP1B1, OAPT1B3, OATP2B1, etc.), organic anion transporters (OAT1, OAT2, OAT3, OAT4, etc.), as well as by the sodium-coupled cotransporters Na^+^/taurocholate co-transporting polypeptide (NTCP) and sodium-dependent organic anion transporter (SOAT) (14, 15). DHEAS can be effluxed by multidrug-resistance proteins (MRP1, MRP2, MRP3, MRP4, etc.) and the breast cancer-resistance protein (BCRP) (6, 16).

Because of its longer half-life (10–20 h) and constant serum concentration, DHEAS is more frequently measured than DHEA (1–3 h) (17, 18). DHEA(S) secretion is regulated by adrenocorticotropic hormone (ACTH). Between the ages of 6 and 10 years, DHEA production gradually starts to increase. This phenomenon is called adrenarche. Peak DHEA(S) serum concentrations are reached in women at about 24 years, followed by a steady decline to around 20% of the peak value (19).

A modest increase of DHEA and DHEAS above the age-related range is observed in premature adrenarche. Significantly increased DHEA and DHEAS are observed in congenital adrenal hyperplasia. Very high DHEA and DHEAS serum levels are characteristic of adrenal tumors with autonomous hormone production (20).

### Case Presentation

An 18.5-year-old female was presented to the outpatient clinic due to overweight. Her height was 166.1 cm, her weight was 80.1 kg, and her body mass index (BMI) was 29.0 kg/m^2^. No clinical signs of hyperandrogenism or hypercortisolism were observed. The skin was normal; however, striae cutis distensae were seen. Neither hirsutism nor acanthosis nigricans was present. No indication of ichthyosis was noted. Pubertal development was complete, with breast stage B5, pubic hair stage PH5, menstrual periods were regular. Blood pressure was within the norm, at 125/80 mm Hg.

The patient fulfilled only two criteria for metabolic syndrome in adolescence by having disturbed glucose metabolism and abdominal obesity. Homeostatic Model Assessment of Insulin Resistance (HOMA-IR) was increased to 6.9; oral glucose tolerance was pathological (2 h glucose concentration 165 mg/dl). Serum concentrations of high-density lipoprotein (HDL)-cholesterol (60 mg/dl) and triglycerides (108 mg/dl) were within the reference range, while cholesterol (232 mg/dl) and low-density lipoprotein (LDL)-cholesterol (186 mg/dl) were increased. The patient underwent a relatively successful lifestyle intervention, which enabled a stabilization of her BMI and a normalization of her oral glucose tolerance within 6 months.

Routine analysis of adrenal hormones revealed an extremely high serum DHEAS level, at 7,546 ng/ml (reference < 4,000; Table 1). A second blood sample confirmed the DHEAS excess, with even a higher serum level, at 8,835 ng/ml. In contrast, serum DHEA was in the normal range, at 443 ng/dl (reference < 750). Serum ACTH (4.8 pmol/L), cortisol (22.7 μg/dl), androstenedione (10 nmol/L), 17-OH progesterone (70 ng/dl), testosterone (28 ng/dl), luteinizing hormone (LH; 5.7 UI/L), follicle-stimulating hormone (FSH; 1.7 IU/L), as well as thyroid-stimulating hormone (TSH; 1.57 mIU/L), and free thyroxine (fT4; 14 pmol/L) levels were all within the normal range.

**Table 1:** Patient's serum hormone data as detected by immunoassay.

**Serum analyte**	**Unit**	**Value**	**Reference range** **(21)**
DHEAS basal	ng/ml	7,546; 8,835^*^	1,450–4,000
After dexa suppression	ng/ml	1,199	
DHEA basal	ng/dl	443	200–750
After dexa suppression	ng/dl	144	
Androstendione	ng/ml	2.9	<3.44
Testosterone	ng/dl	28	<45
ACTH	pg/ml	21.8	<50

* *Range of four measurements*.

*ACTH, adrenocorticotropic hormone; DHEAS, dehydroepiandrosterone sulfate*.

The serum steroid sulfatome was analyzed by liquid chromatography-tandem mass spectrometry (LC-MS/MS). The high DHEAS serum level was confirmed. Interestingly, with the exception of cholesterol sulfate, several other sulfated steroids were found to be above the reference range, namely, pregnenolone sulfate, 17-hydroxypregnenolone sulfate, 16-α-hydroxy-DHEAS, androstenediol-3-sulfate, androsterone sulfate, and epiandrosterone sulfate (Table 2). In addition, the *urinary* steroid metabolome was delineated by gas chromatography-mass spectrometry (GC-MS) in a 24 h urinary sample. It revealed excessive excretion of DHEAS metabolites at 200% of the upper limit of the reference interval. Urinary analysis excluded any known enzyme defect of the adrenal steroid synthesis.

**Table 2:** Patient's level of sulfated steroids as detected by LC-MS/MS.

**Compound**	**Concentration** **(ng/ml)**	**Reference range** **(22, 23)**
Cholesterol sulfate	1,171	500–2,000
Pregnenolone sulfate	122	15–90
17-hydroxypregnenolone sulfate	25	2–13
16-α-hydroxy-DHEAS	294	30–180
DHEAS	5,085	800–3,500
Androstenediol-3-sulfate	294	50–275
Androsterone sulfate	3,367	250–1,500
Epiandrosterone sulfate	773	100–500

*DHEAS, dehydroepiandrosterone sulfate; LC-MS/MS, liquid chromatography-tandem mass spectrometry*.

For exclusion of an autonomous hormone secretion by an adrenal or an ovarian tumor, administration of 0.5 mg dexamethasone four times daily on four consecutive days was performed which resulted in an efficient suppression of DHEAS and DHEA as well as their urinary metabolites. On day 5, the DHEAS level decreased to 1,199 ng/ml (13.6% of baseline; reference < 47.2%) and the DHEA level to 144 ng/dl (32% of baseline; reference < 38.9%) (24). In addition, urinary cortisol metabolites were sufficiently suppressed during the dexamethasone test. Ultrasound and MRI scans of the adrenal glands and the ovaries were normal. No further deterioration in the patient's hormonal investigations was noted during the 4 year course of observation.

Following the hypothesis that the high DHEAS level was likely to be hereditary, we sequenced the *STS* gene, which is responsible for the hydrolysis of aryl and alkyl steroid sulfates and catalyzes the conversion of DHEAS to DHEA (Figure 1). Sequence analysis revealed a heterozygous single-base substitution (g.117217G>T) that results in a nonsense mutation at codon 173 (p.G173X). This mutation predicts a truncation of the carboxyl region of the STS enzyme that is implicated in substrate binding. The mutant allele contained two further variants within the fifth exon: a silent mutation (g.117309C>A) at codon 203 C → A (leucine), and a missense mutation at codon 464 G → T, which resulted in substitution of methionine for isoleucine (g.117465G>T). This nonsense mutation is not present in the Exome Variant Server. At 19 bp upstream in the genomic sequence of exon 10, we identified another C → T transition (g.207619C>T). The nonsense mutation p.G173X found in the patient was *de novo* as demonstrated by allele-specific amplification of the wild-type (wt) and mutant sequences (mut) of both parents' DNA samples. No partial gene deletion of the presumably intact allele outside exon 5 was detected by multiplex ligation-dependent probe amplification.

Biological activity of STS was measured in the patient's blood leukocytes after addition of 1,2,6,7-^3^H-DHEAS. In our experimental setting, ~7 nmol DHEA was produced by 1 × 10^6^ leukocytes per 4 h. The mean (SD) STS activity detected in patient's leukocytes was 107% in comparison to those of five healthy age-matched female controls. Therefore, normal hydrolytic activity of STS was revealed in the patient's blood leukocytes.

No clear explanation for the high levels of DHEAS connected to the *STS* gene structure and enzyme activity was found. Therefore, a defect of one or more transporter proteins was suggested (Figure 1). On the one hand, a reduced steroid sulfate efflux activity in the liver would enhance serum levels; on the other hand, impaired steroid sulfate uptake from the circulation into peripheral target cells or hepatocytes would have the same effect. Prominent candidate efflux transporters were MRP2 and BCRP, both highly expressed at the canalicular membrane of hepatocytes and involved in the hepatobiliary elimination of many drugs and some endogenous substrates such as sulfated steroids (16). However, as MRP2 deficiency would typically increase the levels of conjugated bilirubin in the plasma (Dubin–Johnson syndrome) (25) what was not seen in the patient, BCRP was the only candidate carrier for the efflux site. On the uptake site, several OATP and OAT carriers were on the list. However, as most of them show highly overlapping substrate specificities and redundant expression patterns (e.g., OATP1B1, OATP1B3, and OATP2B1 are expressed in the liver) (14), it was supposed that a transport defect of one of these carriers could have been compensated by another member of the same carrier family. Therefore, we decided to select two carriers from the SLC10 carrier family, which are more unique in the sense of substrate specificity and tissue expression and which both show sodium-dependent transport of sulfated steroids (15, 26). In both carriers, genetic variants or mutations were described before with significantly reduced transport activity, associated with increased plasma levels of their substrates (27–30). However, as the determination of DHEAS serum levels is not included in standard diagnostics, elevated DHEAS levels may have been overseen in patients with such carrier defects. Using exon-spanning PCR, all exons of the abovementioned membrane transporters were sequenced. There were no relevant variants detected for the *SLC10A1* and *SLC10A6* genes. Sequence analysis revealed a heterozygous Q141K variant for BCRP. Interestingly, this variant has in its homozygous state previously been associated with reduced efflux transport activity (31).

## Materials and Methods

### Genomic DNA Extraction and Purification

After written informed consents were obtained from both the parents and the patient, genomic DNA was extracted from 2 ml ethylenediaminetetraacetic acid (EDTA) blood using the NucleoSpin Blood L kit (Macherey-Nagel, Dueren, Germany) following the manufacturer's instructions.

### Polymerase Chain Reaction and Cloning and Sequencing

Exons and intronic flanking regions of genomic DNA were amplified by PCR. Primer sequences and annealing temperatures are available upon request. PCR conditions for amplification of the *STS* gene consisted of an initial period of denaturation at 95°C for 2 min, followed by 40 cycles consisting of 30 s of denaturation at 95°C, 30 s of annealing at 59–65°C, 60 s of extension at 72°C, and a final period of extension at 72°C for 10 min. PCR products were subcloned into pGEM-T easy (Promega, Mannheim, Germany) and sequenced by Sanger sequencing (GATC, Konstanz, Germany). In the case of BCRP, NTCP, and SOAT, the Phusion Flash PCR Master Mix (Thermo Scientific, Waltham, Massachusetts, USA) was used for PCR amplification with the following touch-down protocol: initial denaturation at 98°C for 10 s; 10 cycles of 98°C for 1 s, annealing at Tm + 5°C for 5 s minus 0.5°C each cycle, and 72°C for 15 s; 30 cycles of 98°C for 1 s, annealing at Tm for 5 s, and 72°C for 15 s; and a final extension of 72°C for 1 min. PCR products were directly sequenced after gel extraction and purification with the HiYield Gel/PCR DNA Fragments Extraction kit (Sued-Laborbedarf GmbH, Gauting, Germany) by Sanger sequencing (SeqLab Sequence Laboratories GmbH, Göttingen, Germany).

### Multiplex Ligation-Dependent Probe Amplification Assay

Multiplex ligation-dependent probe amplification (MLPA) (MRC-Holland, Amsterdam, Netherlands) was performed to exclude a partial gene deletion of the presumably intact allele outside exon 5. The SALSA MLPA P160-A2 STS probemix kit was applied in the experiments. Sample DNA denaturation, probe hybridization, probe ligation, and amplification of ligated probes were performed according to the manufacturer's instructions. Amplified probes were analyzed by polyacrylamide gel electrophoresis on a DNA sequencer (Li-Cor Global Edition IR^2^, Long Readir 4200, Li-Cor Biosciences GmbH, Bad Homburg, Germany) using the AIDA program.

### Hormone Assays

Serum DHEAS was measured using an automated chemiluminescence assay system (Immulite, Siemens Healthcare Diagnostics Product Ltd., UK). Inter- and intra-assay coefficients were 8.5 and 4.0%, respectively (21). The serum steroid sulfatome was analyzed by LC-MS/MS as described previously (22). Urine steroid metabolome analysis was performed using GC-MS as described elsewhere (23).

### Steroid Sulfatase Activity Assay

#### Leukocyte Sonicate

STS activity assay was performed as previously described with minor modifications (32). Briefly, peripheral blood was collected in an EDTA syringe. Eight milliliters EDTA-blood were mixed with 2 ml dextran solution, containing 5.0 g Dextran 250 (Roth, Karlsruhe, Germany), 0.7 g NaCl, and 50 mg Na-heparin dissolved in 100 ml bi-distilled water and let stay at room temperature for 1 h. The supernatant, containing leukocytes, was centrifuged at 400 g for 5 min. The pellet was washed with 15 ml phosphate buffered saline (PBS; Gibco by Life Technologies, Paisley, UK), centrifuged at 400 g for 5 min, dissolved in the appropriate amount of PBS to give a final concentration of 5 × 10^6^ leukocytes/100 μl cell suspension and kept at −20°C until further use. After thawing, the leukocyte suspension was sonicated by an ultrasonic processor (Sonifier B-12 cell disruptor, Branson Sonic Power Company, Danbury, Connecticut) three times per 10 s, the procedure being performed on ice to avoid overheating.

#### Steroid Sulfatase Activity

Steroid sulfatase activity was determined using 1,2,6,7-^3^H-DHEAS, sodium salt as a substrate (specific activity 70.5 Ci/mmol; PerkinElmer, Inc., Boston, MA) as previously described (32) with minor modifications. Briefly, 1,2,6,7-^3^H-DHEAS in ethanol was evaporated to dryness and dissolved in 0.1 M Tris buffer, pH 7.6, to yield a final concentration of 0.36 μM in the assay mixture. One hundred microliters 1,2,6,7-^3^H-DHEAS in buffer and 100 μl leukocyte homogenate from 5 × 10^6^ cells were incubated at 37°C for 4 h. The reaction was stopped by adding 3.0 ml diethyl ether, vortexed twice for 30 s, and centrifuged at 350 g for 5 min for phase separation. The organic phase at 1.5 ml was decanted into a scintillation vial, evaporated to dryness, dissolved in 5 ml scintillation fluid, and counted in a β-scintillation counter (β-Counter TricCarb 2900 TR, Canberra-Packard, Frankfurt, Germany). STS activity was examined in three individual experiments, each sample being performed in duplicate. Non-enzymatic activity was subtracted using blank samples in which the reaction was stopped with diethyl ether directly after adding radioactivity to the leukocyte homogenate. Steroid sulfatase activity is expressed as nanomoles DHEA generated per 1 × 10^6^ leukocytes per 4 h. Results for the patient's leukocytes (mean ± SD) are presented as a percent of the activities of the healthy controls.

#### Controls

Five healthy female controls aged 19.1–22.2 years with DHEAS levels within the normal range (1,397–3,057 ng/ml) took part in the study. STS activity assay was performed after written informed consent was obtained.

## Discussion

DHEAS is routinely measured in our unit concerning patients with obesity. The increased levels of DHEAS were found not because of a specific symptom presented by the patient. No clinical signs of hyperandrogenemia were expected to be present, as the active precursor for androgen synthesis, DHEA, was within the normal range.

Our hypothesis explaining the DHEAS excess was the presence of an ovarian or a suprarenal DHEAS-producing tumor. This suspicion was not confirmed. Neither the sonographic and the Magnetic Resonance Tomography (MRT) images nor the effective dexamethasone suppression of both DHEA and DHEAS supported its presence.

STS deficiency was another plausible diagnosis in spite of the complete lack of ichthyosis skin changes. Our findings that the patient had no increased levels of DHEA, and respectively no androgen excess, are in line with a previous study which implies that STS exerts no systemic effect on male sex hormone synthesis from DHEAS (33). It was observed that intravenous (i.v.) infusion of DHEAS in healthy young men did not result in an increase of circulating DHEA. However, in the case of diminished STS activity, serum DHEA and testosterone would have been below the lower limit of the norm (33). The detected nonsense mutation in the *STS* gene, however, was in the heterozygous state; therefore, it was not supposed to be responsible for STS deficiency. In line with the genetic data, the bioassay revealed normal enzyme activity in the patient's leukocytes.

DHEA levels were not decreased, as it might be expected in the case of elevated sulfotransferase activity. Sulfotransferases are relatively substrate specific, and an assumed augmented activity of SULT2A1 could not explain the elevated levels of other sulfated steroids (6). Therefore, it seems not viable that the patient has increased sulfotransferase activity neither due to an increased gene copy number nor to an activating mutation which has not been observed so far.

Not only DHEAS but also other sulfated steroids had increased serum levels in the patient. We searched for a defect in a steroid sulfate carrier protein. However, not all known candidate carriers were investigated in the study. It was only concentrated on the efflux carrier BCRP and the uptake carriers NTCP and SOAT, from which the genomic coding regions were sequenced in the patient (Figure 1). The idea to investigate cellular transporters for sulfated steroids was to search not only for the mechanism behind the elevated serum levels of DHEAS but for a broad range of sulfated steroid molecules. For example, SOAT is capable of transporting pregnenolone sulfate, androsterone sulfate, epiandrosterone sulfate, estrone-3-sulfate, 17β-estradiol-3-sulfate, 17β-estradiol-17-sulfate, and testosterone sulfate in addition to DHEAS (34). Therefore, a carrier defect could explain the serum increase of DHEAS as well as other sulfated steroids found in the patient's serum. However, there was no genetic variant present in the SLC10A1 and SLC10A6 genes, supposing fully active steroid sulfate uptake via NTCP and SOAT. Interestingly, the variant Q141K was found for BCRP in a heterozygous form, and this variant was previously reported to decrease the transport function of BCRP (31, 35). This was shown for the drugs sulfasalazine, topotecan, allopurinol, statins, etc. (35). In the patient presented here, this heterozygous Q141K BCRP variant may contribute to the elevated serum levels of sulfated steroids by their reduced elimination into bile via BCRP. However, it has to be emphasized that the Q141K polymorphism of BCRP is often seen in humans in a heterozygous state. Thus, it is relatively unlikely that this finding alone can lead to the increased serum levels of DHEAS. On the other hand, it is not known how often DHEAS levels are measured in subjects carrying the Q141K polymorphism in the *ABCG2* gene. Furthermore, with a focus on the drug transport function of BCRP, the medications mentioned above should be prescribed to our patient with special care and close monitoring of potential adverse reactions due to decreased elimination.

## Conclusion

The detected novel heterozygous mutation might at least partially inhibit DHEAS (as well as other sulfated steroids) conversion probably due to tissue-specifically decreased STS activity. No defect could be detected in the steroid sulfate uptake carriers NTCP and SOAT. However, as a limitation of the present study, OATP and OAT carriers, also active in steroid sulfate transport, have not been analyzed. The functionally relevant BCRP polymorphism Q141K was detected in a heterozygous state. As a hypothesis, the combination of this heterozygous BCRP polymorphism and the heterozygous nonsense mutation in the *STS* gene may explain the observed high levels of sulfated steroids in our patient.

## Data Availability Statement

The datasets generated for this study are available on request to the corresponding author.

## Ethics Statement

The patient and her parents have given their written informed consent to publish the case.

## Author Contributions

DI performed the STS activity assay and wrote the manuscript. RB was one of the treating physicians of the patient in the outpatient clinic. MH, SW, and AS-G performed the serum steroid sulfatome and urine steroid metabolome analysis and had a big role in writing the manuscript. DH performed the DNA extraction and purification, PCR, cloning and sequencing, and the MLPA assay of the *STS* gene. GBr designed the STS activity assay. AR, GG, and JG performed the molecular genetic analysis of the steroid sulfate transporter proteins and had a big role in writing the manuscript. GBi was one of the treating physicians of the patient in the outpatient clinic, he inspired the study, coordinated und supervised the whole research, and had a substantial role in writing the manuscript. All of the coauthors communicated closely and actively in fruitful discussions throughout the diagnosis and treatment of the patient and took part in the debate over the manuscript, read and approved the submitted version.

## Conflict of Interest

The authors declare that the research was conducted in the absence of any commercial or financial relationships that could be construed as a potential conflict of interest.
